# Long-term prognosis in patients with acute myocardial infarction and newly detected glucose abnormalities: predictive value of oral glucose tolerance test and HbA1c

**DOI:** 10.1186/s12933-021-01315-5

**Published:** 2021-06-14

**Authors:** Stelios Karayiannides, Catarina Djupsjö, Jeanette Kuhl, Claes Hofman-Bang, Anna Norhammar, Martin J. Holzmann, Pia Lundman

**Affiliations:** 1 Department of Clinical Sciences, Karolinska Institutet, Danderyd Hospital, SE-182 88 Stockholm, Sweden; 2 Centre for Diabetes, Academic Specialist Centre, Region Stockholm, Sweden; 3 Department of Medicine (K2), Karolinska Institutet, Stockholm, Sweden; 4 Heart, Vascular and Neuro Theme, Karolinska University Hospital, Stockholm, Sweden; 5 Division of Medicine, Danderyd University Hospital, Stockholm, Sweden; 6 Department of Cardiology, Danderyd University Hospital, Stockholm, Sweden; 7 Capio S:t Görans Hospital, Stockholm, Sweden; 8 Theme of Emergency and Reparative Medicine, Karolinska University Hospital, Stockholm, Sweden

**Keywords:** Myocardial infarction, Diabetes, Prediabetes, Oral glucose tolerance test, Hemoglobin A1c, Prognosis

## Abstract

**Background:**

Disturbances of glucose metabolism can be diagnosed by an oral glucose tolerance test (OGTT) and by glycated haemoglobin (HbA1c). The aim of this study was to investigate the association between newly detected disturbances of glucose metabolism and long-term prognosis after acute myocardial infarction (AMI) and to compare the predictive value of an OGTT and HbA1c.

**Methods:**

Patients under the age of 80 years with no known history of diabetes admitted for AMI at the Department of Cardiology, Danderyd University Hospital, Stockholm, Sweden, from January 1st, 2006 until December 31st, 2013, were investigated with an OGTT and a HbA1c before discharge and were classified as having normal glucose tolerance (NGT), prediabetes or diabetes according to American Diabetes Association (ADA) criteria. Using nationwide, all-inclusive registers, patients were followed for the incidence of combined event [CE (first of myocardial infarction, heart failure, ischaemic stroke or mortality)] for a mean follow-up time of 4.8 years. Cox regression analysis was used to calculate Hazard Ratios (HR) and their 95% confidence intervals (CI).

**Results:**

Of the 841 patients who were investigated with both an OGTT and a HbA1c, 139 (17%) patients had NGT, 398 (47%) had prediabetes and 304 (36%) had diabetes according to OGTT. The corresponding figures using HbA1c were 320 (38%), 461 (55%) and 60 (7%). Patients with newly discovered diabetes were older and had a higher body mass index compared to those with NGT. OGTT was not predictive for CE. In contrast, prediabetes identified by a HbA1c was associated with an increased risk for CE (HR 1.31; 95% CI 1.05–1.63) compared to normoglycaemia. When comparing the prognostic value of different glucose and HbA1c cut-offs, only a HbA1c ≥ 39 mmol/mol was significantly associated with CE (HR 95% CI; 1.30:1.05–1.61).

**Conclusion:**

In this single-centre study, in a recent contemporary cohort, we found that around two thirds of the patients admitted with AMI with no known history of diabetes had disturbed glucose metabolism, in accordance with previous studies. HbA1c in the prediabetes range, but not OGTT, added predictive value on the long-term outcome, in a cohort to whom a pathologic OGTT result was communicated with lifestyle advice.

**Supplementary Information:**

The online version contains supplementary material available at 10.1186/s12933-021-01315-5.

## Background

Diabetes mellitus is an established risk factor for cardiovascular disease and mortality and patients with diabetes have a worse outcome following acute coronary events [1–3]. After acute myocardial infarction (AMI), patients with both known diabetes as well as newly detected abnormal glucose metabolism, have been shown to have a poorer long-term outcome with increased risk for mortality and future cardiovascular events than patients with normal glucose metabolism [4–6]. This may reflect the fact that patients with diabetes are undiagnosed for many years and present with a cardiovascular complication, but could also indicate that the increased cardiovascular risk associated with elevated glucose levels begins before the diagnosis of diabetes [7].

Disturbances of glucose metabolism can be detected by different methods. The oral glucose tolerance test (OGTT) has been the golden standard for many years [8] and haemoglobin A1c (HbA1c) was approved as diagnostic tool in 2009 [9]. Diagnostic criteria for glucose disturbances differ slightly between American Diabetes Association (ADA) and World Health Organisation (WHO) with regards to the cut-off for impaired fasting glycaemia (IFG) and the prediabetes cut-off for HbA1c [10]. To detect prediabetes and diabetes, WHO recommends performing an OGTT, while ADA recommends using a HbA1c, partly because it is less time consuming and therefore more cost-effective. However, there are indications that a HbA1c and an OGTT identify different populations with different risks, [11, 12] and it is yet not clarified if one method is superior to the other in predicting long-term prognosis. Shahim et al., showed that a 2-h postload OGTT glucose (2 h-PG) was a better prognostic test regarding cardiovascular events in patients with established coronary artery disease, but with a mean follow-up time of only 2 years [13]. Another study found that 2 h-PG was an indicator of a poorer prognosis in people in general, but in this study no comparison to HbA1c or outcome in cardiovascular patients was specifically made [14]. The latest European Society of Cardiology guidelines on diabetes, pre-diabetes and cardiovascular diseases state that there is a gap in evidence with regards to whether HbA1c or an OGTT is the best prognostic marker regarding important outcomes in patients with coronary heart disease [15].

The aim of the present study was to investigate the association between newly detected disturbances of glucose metabolism and long-term prognosis after AMI and to compare the predictive value of an OGTT and a HbA1c.

## Methods

### Study design

This was a single-centre, observational, cohort study. An individual consent for inclusion in the study was not collected. The Swedish Patient Data Act allows the inclusion of personal data in a registry for the purpose of improving the quality of care, as long the individual is informed and has the right of opting out at any time and having his/her personal data erased.

The study complied with the Declaration of Helsinki and was approved by the regional research ethics committee in Stockholm, Sweden (reg. no 2014/338-31/2 and 2018/1321-32).

### Study population and data sources

Following publications from Sweden indicating a high proportion of glucose disturbances in patients with AMI [16], the Department of Cardiology at Danderyd University Hospital in Stockholm, Sweden, initiated an implementation program to identify undetected dysglycaemia and offered lifestyle advice and follow-up of these patients. From January 1st, 2006 until December 31st, 2013, 3417 patients with acute coronary syndrome under the age of 80 years were admitted to the Department of Cardiology at Danderyd University Hospital. As part of the usual clinical care, the majority of patients with no known history of diabetes were screened for undiagnosed glucose disturbances with a standardised 75-g OGTT after an overnight fast, with plasma glucose values analysed at 0 and 120 min, no earlier than four days after admission. HbA1c was not routinely taken simultaneously in all patients, particularly at the beginning of the study period. Using information from the Swedish Web-system for Enhancement and Development of Evidence-based care in Heart disease Evaluated According to Recommended Therapies (SWEDEHEART), [17] a national quality registry that contains data on all patients with acute coronary syndromes, we first identified all the patients that were admitted within our inclusion period. Patient records were then examined with regards to OGTT and HbA1c values. We excluded 433 AMI patients with a known history of diabetes and 1300 patients who were not screened for diabetes, most of them due to clinical reasons. In total, 1684 patients with AMI were screened with an OGTT and 841 (50%) of them also had a registered HbA1c value, which was analysed concurrently with the OGTT (Additional file 1: Figure S1). These 841 patients made up our study cohort.

By using the unique personal identity number [18] that every Swedish resident has, data regarding comorbidities, medications and all-cause mortality were collected from four different sources: (1) Swedish Web-system for Enhancement and Development of Evidence-based care in Heart Disease Evaluated According to Recommended Therapies (SWEDEHEART) register, which contains information on patients admitted at Swedish hospitals with acute coronary syndrome and of all the patients undergoing coronary angiography, percutaneous coronary intervention or coronary artery bypass grafting, (2) the National Patient Register (NPR), which includes information on diagnoses according to the ICD-10 system for both inpatient care and outpatient specialist visits, (3) the Prescribed Drug Register, which contains data on all filled prescriptions since July 1st 2005 and (4) the Cause of Death Register, which includes information on date and cause of death for all Swedish residents.

All patients were followed up for all-cause mortality until December 25th, 2017 and for hospitalisation for myocardial infarction, ischaemic stroke or heart failure until December 31st, 2014. We report on the incidence of combined event [CE (first of myocardial infarction, heart failure, ischaemic stroke or mortality)]. The mean follow-up time for our study population was 4.8 years. Information on dispensed prescription medications were collected from 5 months before hospitalisation with AMI to 1 month after discharge.

### Definitions

Based on the results of the OGTT, patients were categorised into three groups according to ADA criteria: [19] (1) Normoglycaemia (NGT), (2) Prediabetes [Impaired glucose tolerance (IGT), Impaired fasting glucose (IFG) or both] and (3) Type 2 diabetes. HbA1c was also used to categorise the patients into three groups according to ADA criteria: (1) Normoglycaemia (NGT), (2) Prediabetes, and (3) Type 2 diabetes (Additional file 1: Table S1). Previously known diabetes was defined as self-reported history in SWEDEHEART. A complete list of all the definitions used in the study can be found in the online supplement (Additional file 1: Table S2).

### Statistical methods

Baseline characteristics are presented as medians with interquartile range for continuous and absolute numbers and percentage for categorical variables. Baseline characteristics stratified by glucose perturbation group were compared using the chi-square or the non-parametric Kruskal–Wallis test as applicable. Time-to-event rates were calculated using survival analysis. Cox regression analysis was used to calculate Hazard Ratios (HR) and their 95% confidence intervals (CI) for all-cause mortality. The proportionality of hazards assumption was not violated when tested using Schoenfeld residuals. A two-sided significance level of p < 0.05 was considered significant for all tests. Statistical analysis was performed using STATA version 14 (STATA Corp., College Station, TX, USA).

## Results

### Baseline characteristics

Baseline characteristics of the 841 patients making up the study population, with OGTT and HbA1c results analysed are shown in Tables 1 and 2.

**Table 1:** Baseline characteristics of our study population stratified by glycaemic status according to OGTT results (ADA criteria)

Characteristic	Available data	NGT	Prediabetes (IFG/IGT)	Diabetes	p-value	Known diabetes
Study population—no known diabetes, n = 841		139 (16.5%)	398 (47.3%)	304 (36.2%)		433
Age—yr [median (IQR)]	841/841	63 (54–69)	64 (58–71)	66 (58–71)	0.019	68 (61–75)
Gender (male)	841/841	104 (74.8%)	304 (76.4%)	212 (69.7%)	0.133	314 (72.5%)
Body-mass index [median (IQR)]	796/841	25 (23–28)	27 (24–30)	28 (25–30)	< 0.001	28 (26–32)
Smokers	801/841	38 (29.5%)	142 (37.3%)	118 (40.5%)	0.054	84 (21.9%)
Snuff users	560/841	2 (2.0%)	23 (8.4%)	12 (6.5%)	0.083	9 (3.5%)
eGFR (mL/min/1.73 m2)—MDRD equation [median (IQR)]	769/841	81 (69–89)	83 (72–97)	83 (70–96)	0.177	73 (55–92)
Medical history						
Previous myocardial infarction	841/841	40 (28.8%)	101 (25.4%)	63 (20.7%)	0.143	103 (23.8%)
Hypertension	841/841	36 (25.9%)	88 (22.1%)	71 (23.4%)	0.658	179 (41.3%)
Heart failure	841/841	3 (2.2%)	5 (1.3%)	3 (1.0%)	0.598	14 (3.2%)
Atrial fibrillation	841/841	8 (5.8%)	14 (3.5%)	13 (4.3%)	0.520	26 (6.0%)
Previous PCI or CABG	841/841	32 (23.0%)	84 (21.1%)	58 (19.1%)	0.612	83 (19.2%)
Stroke	841/841	5 (3.6%)	8 (2.0%)	12 (3.9%)	0.291	23 (5.3%)
Peripheral artery disease	841/841	2 (1.4%)	6 (1.5%)	3 (1.0%)	0.825	13 (3.0%)
Chronic kidney disease	841/841	2 (1.4%)	2 (0.5%)	2 (0.7%)	0.523	14 (3.2%)
Chronic obstructive pulmonary disease	841/841	3 (2.2%)	3 (0.8%)	3 (1.0%)	0.377	8 (1.8%)
Medications on discharge						
Aspirin	841/841	121 (87.1%)	342 (85.9%)	246 (80.9%)	0.121	280 (64.7%)
Clopidogrel	841/841	79 (56.8%)	225 (56.5%)	188 (61.8%)	0.334	190 (43.9%)
Ticagrelor	841/841	44 (31.7%)	95 (23.9%)	46 (15.1%)	< 0.001	56 (12.9%)
Prasugrel	841/841	0 (0%)	4 (1.0%)	3 (1.0%)	0.497	8 (1.9%)
ACE-inhibitors	841/841	89 (64.0%)	257 (64.6%)	189 (62.2%)	0.802	202 (46.7%)
ARB	841/841	25 (18.0%)	65 (16.3%)	54 (17.8%)	0.845	95 (21.9%)
Beta-receptor blockers	841/841	126 (90.7%)	341 (85.7%)	251 (82.6%)	0.08	277 (64.0%)
Statins	841/841	122 (87.8%)	342 (85.9%)	252 (82.9%)	0.338	271 (62.6%)
Dihydropyridines	841/841	22 (15.8%)	70 (17.6%)	69 (22.7%)	0.129	111 (25.6%)
Diuretics	841/841	15 (10.8%)	65 (16.3%)	59 (19.4%)	0.076	101 (23.3%)
Warfarin	841/841	7 (5.0%)	21 (5.3%)	14 (4.6%)	0.921	29 (6.7%)

**Table 2:** Baseline characteristics of our study population stratified by glycaemic status according to HbA1c results (ADA criteria)

Characteristic	Available data	NGT	Prediabetes	Diabetes	p-value	Known diabetes
Study population—no known diabetes, n = 841		320 (38.1%)	461 (54.8%)	60 (7.1%)		433
Age—yr [median (IQR)]	841/841	63 (56–70)	66 (59–71)	66 (57–72)	0.026	68 (61–75)
Gender (male)	841/841	242 (75.6%)	334 (72.5%)	44 (73.3%)	0.610	314 (72.5%)
Body-mass index [median (IQR)]	796/841	26 (24–29)	27 (25–30)	29 (25–32)	< 0.001	28 (26–32)
Smokers	801/841	103 (33.1%)	169 (39.1%)	26 (44.8%)	0.035	84 (21.9%)
Snuff users	560/841	11 (4.9%)	26 (8.9%)	0 (0%)	0.034	9 (3.5%)
eGFR (mL/min/1.73 m2)—MDRD equation [median (IQR)]	769/841	85 (71–96)	81 (71–94)	85 (76–98)	0.104	73 (55–92)
Medical history						
Previous myocardial infarction	841/841	82 (25.6%)	108 (23.4%)	14 (23.3%)	0.769	103 (23.8%)
Hypertension	841/841	72 (22.5%)	110 (23.9%)	13 (21.7%)	0.869	179 (41.3%)
Heart failure	841/841	3 (0.9%)	7 (1.5%)	1 (1.7%)	0.756	14 (3.2%)
Atrial fibrillation	841/841	10 (3.1%)	21 (4.6%)	4 (6.7%)	0.371	26 (6.0%)
Previous PCI or CABG	841/841	64 (20.0%)	99 (21.5%)	11 (18.3%)	0.791	83 (19.2%)
Stroke	841/841	7 (2.2%)	17 (3.7%)	1 (1.7%)	0.395	23 (5.3%)
Peripheral artery disease	841/841	3 (0.9%)	6 (1.3%)	2 (3.3%)	0.325	13 (3.0%)
Chronic kidney disease	841/841	2 (0.6%)	4 (0.9%)	0 (0%)	0.733	14 (3.2%)
Chronic obstructive pulmonary disease	841/841	2 (0.6%)	6 (1.3%)	1 (1.7%)	0.596	8 (1.8%)
Medications on discharge						
Aspirin	841/841	271 (84.7%)	391 (84.8%)	47 (78.3%)	0.418	280 (64.7%)
Clopidogrel	841/841	175 (54.7%)	282 (61.2%)	35 (58.3%)	0.195	190 (43.9%)
Ticagrelor	841/841	92 (28.8%)	82 (17.8%)	11 (18.3%)	0.001	56 (12.9%)
Prasugrel	841/841	2 (0.6%)	5 (1.1%)	0 (0%)	0.599	8 (1.9%)
ACE-inhibitors	841/841	218 (68.1%)	278 (60.3%)	39 (65.0%)	0.08	202 (46.7%)
ARB	841/841	44 (13.8%)	96 (20.8%)	4 (6.7%)	0.003	95 (21.9%)
Beta-receptor blockers	841/841	278 (86.9%)	394 (85.5%)	46 (76.7%)	0.121	277 (64.0%)
Statins	841/841	276 (86.3%)	393 (85.3%)	47 (78.3%)	0.285	271 (62.6%)
Dihydropyridines	841/841	60 (18.8%)	88 (19.1%)	13 (21.7%)	0.870	111 (25.6%)
Diuretics	841/841	49 (15.3%)	80 (17.4%)	10 (16.7%)	0.752	101 (23.3%)
Warfarin	841/841	11 (3.4%)	26 (5.6%)	5 (8.3%)	0.178	29 (6.7%)

### Glucose disturbances by OGTT result

When investigated with an OGTT, a total of 139 (17%) patients had NGT, 398 (47%) had prediabetes (IGT and/or IFG) and 304 (36%) had newly discovered diabetes. Patients with newly discovered diabetes were older and had a higher BMI than patients with NGT and prediabetes. Patients with newly discovered diabetes were significantly less often treated with ticagrelor compared to patients with NGT and prediabetes. There were no significant differences in comorbidities or treatment with aspirin, clopidogrel, ACE-inhibitors, beta-receptor blockers or statins between the three groups.

Baseline characteristics of all patients who underwent an OGTT (n = 1684) are shown in Additional file 1: Table S3.

### Glucose disturbances by HbA1c results

When patients were categorised into groups according to HbA1c results, 320 (38%) patients had a normal HbA1c (< 39 mmol/mol), 461 (55%) had prediabetes (HbA1c 39–47 mmol/mol) and 60 (7%) were defined as having diabetes (HbA1c ≥ 48 mmol/mol). Similar findings were observed as with the OGTT, where patients with newly discovered diabetes were older and had a higher BMI than the other two groups. Those with diabetes were significantly less often treated with angiotensin II receptor-blockers and ticagrelor compared with patients with NGT and prediabetes. We observed no other significant differences in comorbidities or medications between the three groups.

**Fig. 1:**
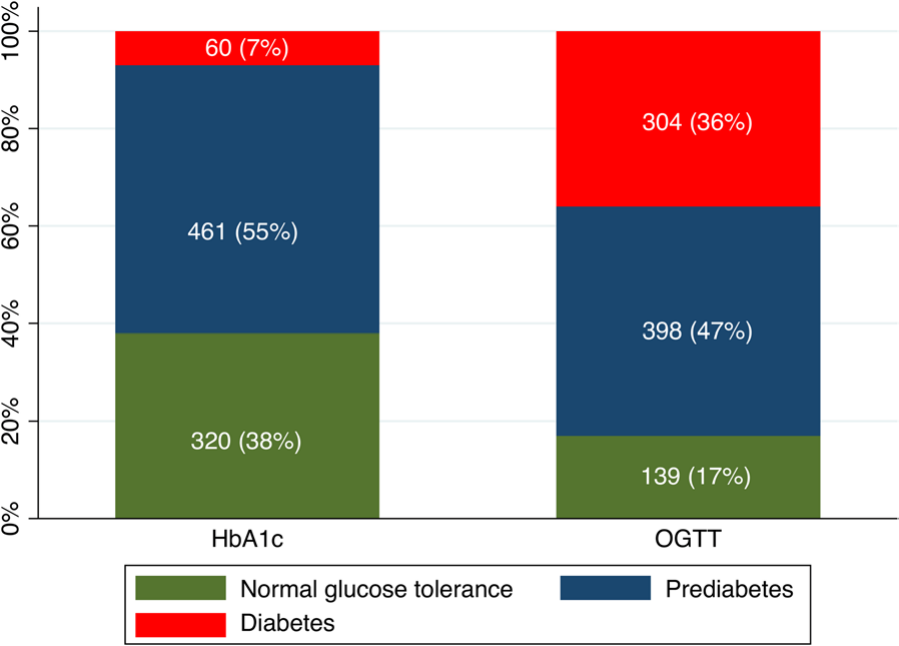
Number and percentage of patients in different glucose groups according to OGTT and HbA1c results (ADA criteria)

The numbers (and percentages) of patients in the different glucose groups according OGTT and HbA1C results are illustrated in Fig. 1.

### Comparison between OGTT and HbA1c results

**Fig. 2:**
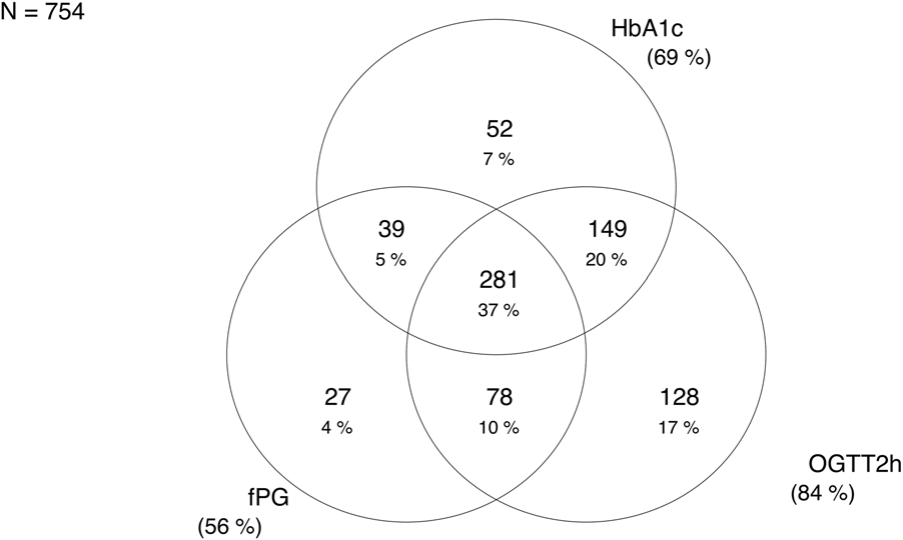
Venn diagram showing proportions and their overlap between fPG, 2 h-PG and HbA1c in classifying dysglycaemia (prediabetes or diabetes) in the 754 out of 841 patients who had dysglycaemia according to either OGTT or HbA1c result (ADA criteria)

Only 87 (10%) out of 841 patients had fasting plasma glucose (fPG), 2 h-PG and HbA1c values within the normoglycaemic range. Of the 754 patients with dysglycaemia (prediabetes or diabetes), 425 (56%) were identified by a single fPG, 636 (84%) by a 2 h-PG and 521 (69%) by a HbA1c. The OGTT (fPG and 2 h-PG) identified 702 patients (93%) as having dysglycaemia and the combination of fPG and HbA1c identified 626 patients (83%). Screening for dysglycaemia with OGTT and HbA1c identified different at-risk groups. Only 281 (37%) patients had values showing dysglycaemia as identified by both fPG, 2 h-PG and HbA1c. The concordance of the OGTT and a HbA1c in classifying dysglycaemia was modest, with only 62% of patients classified as having dysglycaemia on both the OGTT and HbA1c (Fig. 2).

### Event rates and long-term outcome in patient groups according to OGTT results

Of the study population of 841 patients, 108 (12.8%) died during a mean follow-up time of 6.9 years. With regards to cardiovascular events, 175 (20.8%) patients suffered a myocardial infarction, 19 (2.3%) patients had an ischaemic stroke, and 120 (14.3%) patients were hospitalised for heart failure. The total number of combined events (first of myocardial infarction, stroke, heart failure or mortality) was 372 (44.2%).

**Fig. 3:**
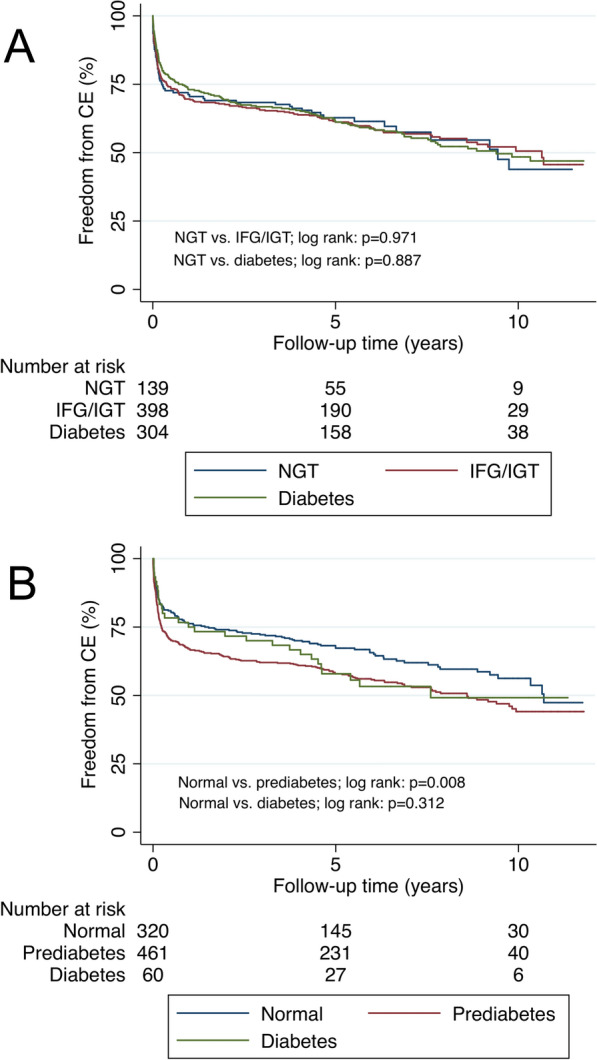
Kaplan–Meier curve showing time to freedom from combined event (CE; first of myocardial infarction, hospitalisation for heart failure, ischaemic stroke or mortality) for different categories of dysglycaemia according to **A** Fasting and 2 h-PG results (OGTT) and **B** HbA1c

Regarding the incidence of CE, 58 (41.7%) patients with NGT had events, 174 (43.7%) events occurred in the prediabetes group and 140 (46.1%) events in the group with patients with newly discovered diabetes. There was no significant difference in hazard ratios neither for the CE nor for all-cause mortality between the three patient groups, both unadjusted and adjusted for age and sex (Table 3 and Additional file 1: Table S4). Figure 3A shows a Kaplan–Meier curve with freedom from CE and Additional file 1: Figure S2A shows a Kaplan–Meier curve with freedom from all-cause mortality between the three groups, categorised according to OGTT results.

**Table 3:** Absolute numbers (%), event rates per 100 person-years and hazard ratios for the combined event (CE; first of myocardial infarction, hospitalisation for heart failure, ischaemic stroke or mortality) in 841 patients stratified by glucose perturbation group according to OGTT and HbA1c results (ADA criteria)

	Total study population	Glycaemic status according to OGTT			Glycaemic status according to HbA1c		
		Normal	Prediabetes	Diabetes	Normal	Prediabetes	Diabetes
	(N = 841)	(N = 139)	(N = 398)	(N = 304)	(N = 320)	(N = 461)	(N = 60)
Number of events, N (%)	372 (44.2)	58 (41.7)	174 (43.7)	140 (46.1)	121 (37.8)	223 (48.4)	28 (46.7)
Events/100 patient-years (95% CI)	9.2 (8.3–10.2)	9.7 (7.5–12.5)	9.3 (8.0–10.7)	9.0 (7.6–10.6)	7.6 (6.4–9.1)	10.3 (9.1–11.8)	9.8 (6.8–14.2)
Unadjusted model		1 (ref)	0.99 (0.73–1.34)	0.98 (0.72–1.34)	1 (ref)	1.35 (1.08–1.68)	1.25 (0.84–1.85)
Age- and sex-adjusted model		1 (ref)	0.94 (0.69–1.26)	0.92 (0.67–1.25)	1 (ref)	1.31 (1.05–1.63)	1.22 (0.81–1.85)

CI: confidence interval; ref: reference category; N: number of patients

Similarly, there were no significant differences in the incidence rates for the CE or HRs between the three patient groups when looking at the total number of patients who underwent an OGTT (n = 1684) (Additional file 1: Table S5).

### Event rates and long-term outcome in patient groups according to HbA1c results

When patients were stratified by glucose groups according to HbA1c results, the CE of first of myocardial infarction, ischaemic stroke, hospitalisation for heart failure or mortality occurred in 121 (37.8%) patients with normoglycaemia (HbA1c < 39 mmol/mol), in 223 (48.4%) patients with prediabetes (HbA1c 39–47 mmol/mol) and in 28 (46.7%) patients with newly discovered diabetes (HbA1c ≥ 48 mmol/mol). Patients with prediabetes had a poorer prognosis, with significantly higher risk for cardiovascular events and mortality. When adjusted for age and sex, the HR for the combined event was 1.31 (CI 1.05–1.63) compared to the group with normal HbA1c (Table 3). Figure 3B illustrates a Kaplan–Meier curve with freedom from the CE between the three patient groups and Additional file 1: Figure S2B illustrates a Kaplan Meier curve with freedom from all-cause mortality, categorised according to HbA1c results.

### Comparison of associated risk for combined event by different glucose and HbA1c thresholds according to ADA and WHO criteria

**Fig. 4:**
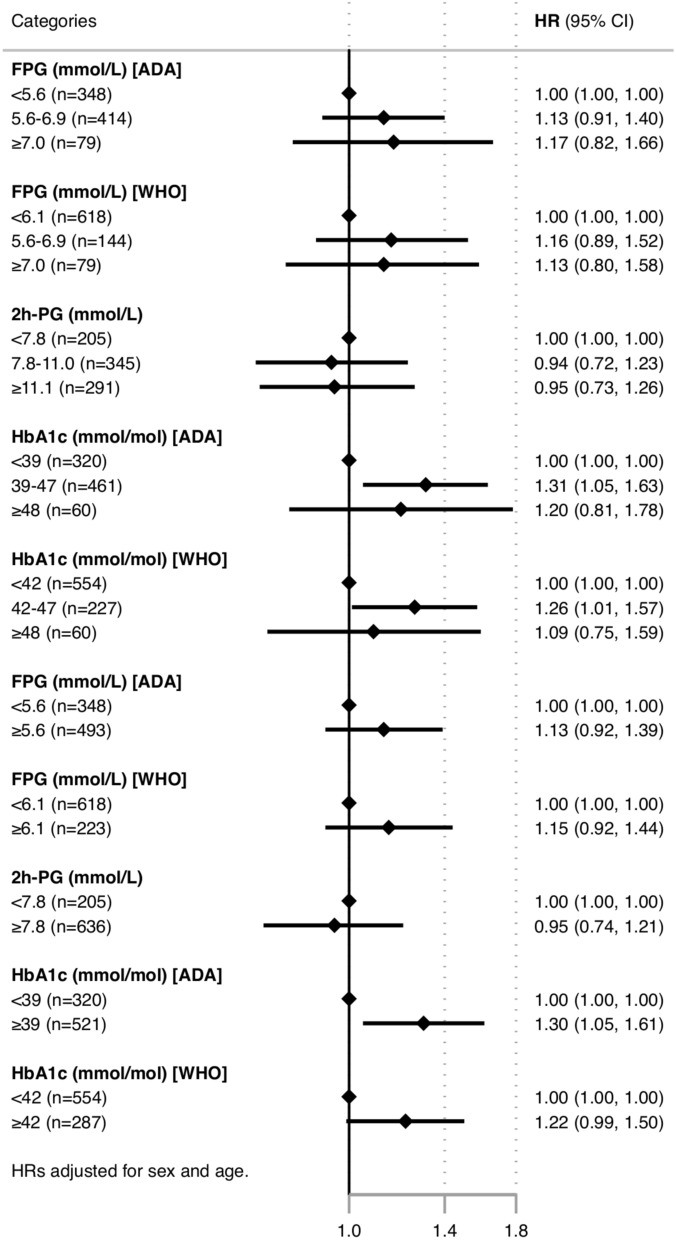
Forest plot with HR (95% CI) for the prognostic value of FPG, 2 h-PG and HbA1c for the combined event (first of mortality, heart failure, ischaemic stroke, and myocardial infarction) using different cut-offs for dysglycaemia according to ADA and WHO definitions

Figure 4 depicts a forest plot with HRs for the CE, adjusted for age and sex, in patients categorised into different glucose groups according to both ADA and WHO criteria. Results are also shown with patients stratified into three or two groups according to fPG, 2 h-PG and HbA1c results. Similar findings are demonstrated, regardless of whether ADA or WHO criteria were used, when patients were divided into three groups, with worse outcomes in the prediabetes group categorised by HbA1c. When patients were divided into two groups (normal or abnormal) there was a statistically significant increased risk for CE only when using the HbA1c cut-off of 39 mmol/mol, i.e., ADA criteria. A similar trend was seen when using the HbA1c cut-off of 42 mmol/mol (WHO), but this did not reach statistical significance.

## Discussion

The present study confirms that approximately two thirds of patients admitted with an AMI have previously undetected glucose disturbances. An OGTT classified 83% of our study population as having dysglycaemia, while a single HbA1c classified 62%. It is well known that a large number of patients with type 2 diabetes remain undiagnosed for many years and that a long duration of undetected hyperglycaemia may have detrimental effects on both micro- and macrovascular complications [20]. By screening for glucose disturbances in patients with AMI, lifestyle modification and pharmacological treatment can be initiated earlier. In a recently published register study, it was shown that patients with prediabetes treated with metformin have an improved outcome [21]. However, whether early lifestyle and medical interventions have a positive effect on short- and/or long-term outcome needs to be further established in randomised intervention studies.

The latest European Society of Cardiology guidelines on diabetes, pre-diabetes and cardiovascular diseases recommend that screening for diabetes in patients with cardiovascular disease should begin with fPG and HbA1c and, only if needed, complemented with an OGTT [15]. This recommendation seems to be supported by the results of our study, as an OGTT (fPG + 2 h-PG) identifies only 10% more patients as having dysglycaemia compared to the combination of fPG and HbA1c. Moreover, an OGTT should be performed no earlier than four days after an AMI to increase its reliability [15]. This is becoming more difficult due to the decreasing length of hospital stay for AMI–Sweden, for example, had an average length of stay of 4.1 days in 2017 [22]. Previous studies have shown that an OGTT is more sensitive in identifying dysglycaemia in populations with coronary artery disease [12, 23] and acute stroke [24]. However, in a population with overweight or obesity, HbA1c identified more patients with prediabetes than an OGTT [25].

As has been noted in other studies, [12, 25] the OGTT and HbA1c seem to identify different high-at-risk populations with a relatively low concordance between them. As such, it is particularly clinically relevant to identify the test which has the most predictive value for important patient outcomes in patients with AMI. Results from previous studies have been conflicting [13, 26, 27]. In our study, HbA1c was a better predictor of future cardiovascular events than either fPG or 2 h-PG. The OGTT did not predict mortality or combined events in any of the glucose groups—neither in the group of patients who had HbA1c simultaneously analysed (n = 841), nor in the larger group of patients who only underwent testing with an OGTT (n = 1684). This finding is in agreement with previous studies that showed that HbA1c was better than fPG in predicting the risk for all-cause mortality and cardiovascular events [26]. However, a report from EUROASPIRE IV, concluded that only 2 h-PG levels and not fPG or HbA1c in patients with stable coronary artery disease predicted future cardiovascular events [13]. Similarly, another study in patients with AMI showed that 2 h-PG, but not fPG was predictive for the risk for all-cause mortality or reinfarction. In this study HbA1c was not studied [27].

Interestingly, when patients were categorised according to HbA1c levels, the group of patients with prediabetes had a higher incidence of death, myocardial infarction, ischaemic stroke and hospitalisation for heart failure than patients who were categorised as having diabetes. Patients with prediabetes (HbA1c 39 to 47 mmol/mol) had a 30% higher risk for the combined outcome compared to patients with normoglycemia (HbA1c < 39 mmol/L). Although point estimates suggested a higher risk for the combined event also in patients with newly detected diabetes (HbA1c ≥ 48 mmol/mol) compared to patients with normoglycaemia, the confidence intervals were wide and the result non-significant.

There might be different explanations for these results. In this observational single-centre study, performed in patients treated with routine clinical care for AMI, there was a high attention and interest in disturbed glucose metabolism and its effects on cardiovascular outcomes. Therefore, screening with an OGTT in patients with AMI as a standard was introduced in 2006. Patients with glucose disturbances, in particular patients with newly detected glucose abnormalities by an OGTT, were offered lifestyle modification counselling and were frequently referred to their general practitioner for a follow-up diagnostic OGTT and ongoing management. This may have resulted in an improved secondary prevention, which may have been superior in patients with diabetes compared to the patients with prediabetes, i.e., patients with diagnosed diabetes were more likely to be treated with guideline-recommended cardiovascular drugs which would lower their risk for adverse outcomes. This intervention was however not performed in a randomised fashion, and we have no information on lifestyle modification achievement or long-term medical treatment.

Another explanation could be that, even though HbA1c was recommended as a diagnostic tool by ADA in 2009^9^ and by WHO in 2011 [28], it was not used routinely in the screening of patients with cardiovascular disease in Sweden until 2014. During the inclusion period for this study, 2006 to 2013, cardiologists were not familiar with considering HbA1c results as a diagnostic tool for type 2 diabetes mellitus, meaning that a pathological OGTT result was more likely to attract attention from the attending physician than a HbA1c in the prediabetic range.

## Strengths and limitations

This study was conducted at one centre and is an observational study and should be reproduced in other hospitals for evaluation of generalisability. Outcomes were retrieved from Swedish national registers, which have been shown to have high validity [29]. There was virtually no loss to follow-up as every individual in Sweden has their own unique personal identity number. We used a standardised method to measure HbA1c and all OGTTs were performed according to WHO guidelines. All samples were analysed in one single accredited laboratory ensuring a high validity of the measures obtained in this study.

We only had information on laboratory values and medical treatment at the time of the index AMI. The effects of lifestyle modification and secondary preventive medical treatment that could affect the outcome could not be analysed. At the beginning of the inclusion period, when HbA1c was not used routinely as a diagnostic tool, most likely a selection bias was introduced, as there was a tendency to have an additional HbA1c analysed in those with a pathologic OGTT. Furthermore, with missing data for a number of variables, we only adjusted data for age and sex, therefore residual confounding may be present.

## Conclusions

In conclusion, we confirm that two thirds of patients with AMI with no previously known history of diabetes have dysglycaemia when investigated with either an OGTT or HbA1c, illustrating the importance of screening for diabetes in this population. In this observational study, only dysglycaemia classified by a HbA1c, but not an OGTT, analysed at the time of the AMI, added predictive value to the long-term prognosis. Our findings seem to support the latest guidelines recommending to primarily use fPG and HbA1c in screening for diabetes after AMI and, only if needed, complement with an OGTT. However, increased awareness of glucose disturbances revealed by an OGTT and a special cardiac rehabilitation program for this group, might have influenced the results.

## Supplementary Information

**Additional file 1: Figure S1.** Flowchart for the study population. **Table S1.** Definitions of normal glycaemic status and dysglycaemia according to ADA and WHO criteria. **Table S2.** List of ICD-10 codes and definitions used in our study. **Table S3.** Baseline characteristics of all patients screened with OGTT (n =1 684) stratified by glycaemic status according to ADA criteria. **Table S4.** Absolute numbers (%), event rates per 100 person-years and hazard ratios for all-cause mortality in 841 patients stratified by glucose perturbation group according to OGTT and HbA1c results (ADA criteria). **Figure S2.** Kaplan–Meier curve showing time to freedom from all-cause mortality for different categories of dysglycaemia according to A. Fasting and 2-hour post-load glucose results (OGTT) and B. HbA1c. **Table S5.** Absolute numbers (%), event rates per 100 person-years and hazard ratios for the combined event (CE; first of myocardial infarction, hospitalisation for heart failure, ischaemic stroke or mortality) in 1684 patients stratified by glucose perturbation group according to OGTT (ADA criteria).

## Data Availability

The datasets used and/or analysed during the current study are available from the corresponding author on reasonable request.

## Abbreviations

OGTT: Oral glucose tolerance test
HbA1c: Haemoglobin A1c
AMI: Acute myocardial infarction
NGT: Normal glucose tolerance
HR: Hazard ratio
CI: Confidence interval
ADA: American Diabetes Association
WHO: World Health Organisation
IFG: Impaired fasting glycaemia
IGT: Impaired glucose tolerance
fPG: Fasting glucose
2 h-PG: 2-Hour postload glucose
CE: Combined event
