# Supplementary material for: Long-term prognosis in patients with acute myocardial infarction and newly detected glucose abnormalities: predictive value of oral glucose tolerance test and HbA1c
Source: Cardiovasc Diabetol. 2021 Jun 14;20:122. doi: 10.1186/s12933-021-01315-5 (PMC8204442; doi:10.1186/s12933-021-01315-5)
Supplement: Supplementary file 1 — Additional file 1: Figure S1. Flowchart for the study population. Table S1. Definitions of normal glycaemic status and dysglycaemia according to ADA and WHO criteria. Table S2. List of ICD-10 codes and definitions used in our study. Table S3. Baseline characteristics of all patients screened with OGTT (n =1 684) stratified by glycaemic status according to ADA criteria. Table S4. Absolute numbers (%), event rates per 100 person-years and hazard ratios for all-cause mortality in 841 patients stratified by glucose perturbation group according to OGTT and HbA1c results (ADA criteria). Figure S2. Kaplan–Meier curve showing time to freedom from all-cause mortality for different categories of dysglycaemia according to A. Fasting and 2-hour post-load glucose results (OGTT) and B. HbA1c. Table S5. Absolute numbers (%), event rates per 100 person-years and hazard ratios for the combined event (CE; first of myocardial infarction, hospitalisation for heart failure, ischaemic stroke or mortality) in 1684 patients stratified by glucose perturbation group according to OGTT (ADA criteria). [file 12933_2021_1315_MOESM1_ESM.docx]

**Long-term prognosis in patients with acute myocardial infarction and newly detected glucose abnormalities: predictive value of oral glucose tolerance test and HbA1c**

**Stelios Karayiannides, MD ^1,2^*, Catarina Djupsjö, MD ^3,4^*, Jeanette Kuhl MD, PhD^3,5^, Claes Hofman-Bang^6^, Anna Norhammar MD, PhD^3,7^, Martin J Holzmann MD, PhD^3,8^, Pia Lundman MD, PhD ^1,6^**

*These authors contributed equally to this work

^1^Department of Clinical Sciences, Karolinska Institutet Danderyd Hospital, Stockholm, Sweden

^2^Centre for Diabetes, Academic Specialist Centre, Region Stockholm, Sweden

^3^Department of Medicine (K2), Karolinska Institutet, Stockholm, Sweden

^4^Heart, Vascular and Neuro Theme, Karolinska University Hospital, Stockholm, Sweden

^5^Division of Medicine, Danderyd University Hospital, Stockholm, Sweden

^6^Department of Cardiology, Danderyd University Hospital, Stockholm, Sweden

^7^Capio S:t Görans Hospital, Stockholm, Sweden

^8^Theme of Emergency and Reparative Medicine, Karolinska University Hospital, Stockholm, Sweden

**Supplementary material**

**Figure S1:** Flowchart for the study population. **Table S1:** Definitions of normal glycaemic status and dysglycaemia according to ADA and WHO criteria. **Table S2:** List of ICD-10 codes and definitions used in our study. **Table S3:** Baseline characteristics of all patients screened with OGTT (n=1 684) stratified by glycaemic status according to ADA criteria. **Table S4:** Absolute numbers (%), event rates per 100 person-years and hazard ratios for all-cause mortality in 841 patients stratified by glucose perturbation group according to OGTT and HbA1c results (ADA criteria). **Figure S2**: Kaplan–Meier curve showing time to freedom from all-cause mortality for different categories of dysglycaemia according to A. Fasting and 2-hour post-load glucose results (OGTT) and B. HbA1c. **Table S5:** Absolute numbers (%), event rates per 100 person-years and hazard ratios for the combined event (CE; first of myocardial infarction, hospitalisation for heart failure, ischaemic stroke or mortality) in 1684 patients stratified by glucose perturbation group according to OGTT (ADA criteria).

**Figure S1:** Flowchart for the study population.

**Table S1:** Definitions of normal glycaemic status and dysglycaemia according to ADA and WHO criteria.

|  | **Glucose (mmol/L)** | | **HbA1c (mmol/mol)** |  |
| --- | --- | --- | --- | --- |
|  | **fPG** | **2h-PG** |  |  |
| Normal glycaemic status | <5.6 (ADA) <6.1 (WHO) | <7.8 | <39 (ADA) <42 (WHO) |  |
|  |  |  |  |  |
| Impaired fasting glycaemia (IFG) | 5.6–6.9 (ADA) 6.1–6.9 (WHO) | <7.8 |  |  |
| Impaired glucose tolerance (IGT) | <5.6 (ADA) <6.1 (WHO) | 7.8–11.0 |  |  |
| Prediabetes |  |  | 39–47 (ADA) 42–47 (WHO) |  |
|  |  |  |  |  |
| Type 2 diabetes | ≥7.0 | ≥11.1 | ≥48 |  |
|  |  |  |  |  |

**Table S2:** List of ICD-10 codes and definitions used in our study.

|  | | **ICD-10 code**  **beginning with** |  |
| --- | --- | --- | --- |
| Myocardial infarction | as outcome | I21, I22, I25 | Primary diagnosis |
|  | in history | I21, I22, I25 | Any diagnosis |
| Heart failure | as outcome | I50, I11.0, I13.0, I13.2, I25.5, K76.1, I42-43 | Primary diagnosis |
|  | in history | I50, I11.0, I13.0, I13.2, I25.5, K76.1, I42-43 | Any diagnosis |
| Ischaemic stroke | as outcome | I63, I61 | Primary diagnosis |
|  | in history | I63, I61 | Any diagnosis |
| Diabetes mellitus | as outcome | See Table 1 |  |
|  | in history | E10-14 | Any diagnosis |
| Hypertension | | I10-15 | Any diagnosis |
| Previous PCI | | FNG02, FNG05 | Any diagnosis |
| Peripheral arterial disease | | I70-73 | Any diagnosis |
| CABG | | Z95.1, FNA00, FNA10, FNC10, FNC20, FNC30, FNC40, FNG00 | Any diagnosis |
| Peripheral artery disease | | I70, I73 | Any diagnosis |
| COPD | | J44 | Any diagnosis |
| Chronic kidney disease | | N18 | Any diagnosis |
| **Definitions used in our study** | | |  |
| Index date | | Date of admission at Cardiology Department, Danderyd Hospital. | |
| Previous comorbidities | | Previous diagnoses in the patient register up to the index date. | |
| Medication at baseline | | Based on the presence of a filled prescription within 5 months before and up to one month after index date. | |
| Combined Events | | Mortality or first hospitalisation for ischaemic stroke, myocardial infarction, or incident heart failure occurring after the index date. | |
|  | |  |  |

**Table S3:** Baseline characteristics of all patients screened with OGTT (n=1 684) stratified by glycaemic status according to ADA criteria.

| **Characteristic** | **Available  data** | **NGT** | **Prediabetes  (IFG/IGT)** | **Diabetes** | **p-value** | **Known  diabetes** |  |
| --- | --- | --- | --- | --- | --- | --- | --- |
| Study population not known diabetes, n= 1 684 |  | 538 (32.0%) | 816 (48.5%) | 330 (19.6%) |  | 433 |  |
| Age – yr [median (IQR)] | 1684/1684 | 63 (54-70) | 65 (57-72) | 66 (59-71) | <0.001 | 68 (61-75) |  |
| Gender (male) | 1684/1684 | 390 (72.5%) | 618 (75.7%) | 229 (69.4%) | 0.073 | 314 (72.5%) |  |
| Body-mass index [median (IQR)] | 1590/1684 | 25 (23-28) | 27 (24-29) | 28 (25-30) | <0.001 | 28 (26-32) |  |
| Smokers | 1605/1684 | 195 (38.2%) | 273 (35.0%) | 130 (41.4%) | 0.081 | 84 (21.9%) |  |
| Snuff users | 1054/1684 | 27 (8.3%) | 45 (8.5%) | 12 (6.0%) | 0.766 | 9 (3.5%) |  |
| eGFR (mL/min/1.73 m2) - MDRD equation [median (IQR)] | 1523/1684 | 81 (72-93) | 82 (71-95) | 83 (69-95) | 0.843 | 73 (55-92) |  |
|  |  |  |  |  |  |  |  |
| ***Medical history*** |  |  |  |  |  |  |  |
| Previous myocardial  infarction | 1684/1684 | 127 (23.6%) | 190 (23.3%) | 69 (20.9%) | 0.619 | 103 (23.8%) |  |
| Hypertension | 1684/1684 | 101 (18.8%) | 173 (21.2%) | 77 (23.3%) | 0.259 | 179 (41.3%) |  |
| Heart failure | 1684/1684 | 11 (2.0%) | 17 (2.1%) | 4 (1.2%) | 0.593 | 14 (3.2%) |  |
| Atrial fibrillation | 1684/1684 | 17 (3.2%) | 41 (5.0%) | 16 (4.9%) | 0.236 | 26 (6.0%) |  |
| Previous PCI or  CABG | 1684/1684 | 108 (20.1%) | 151 (18.5%) | 64 (19.4%) | 0.768 | 83 (19.2%) |  |
| Stroke | 1684/1684 | 12 (2.2%) | 13 (1.6%) | 12 (3.6%) | 0.102 | 23 (5.3%) |  |
| Peripheral  artery disease | 1684/1684 | 10 (1.9%) | 11 (1.4%) | 4 (1.2%) | 0.675 | 13 (3.0%) |  |
| Chronic kidney disease | 1684/1684 | 5 (0.9%) | 2 (0.3%) | 2 (0.6%) | 0.235 | 14 (3.2%) |  |
| Chronic obstructive  pulmonary disease | 1684/1684 | 6 (1.1%) | 9 (1.1%) | 3 (0.9%) | 0.951 | 8 (1.8%) |  |
|  |  |  |  |  |  |  |  |
| ***Medication at discharge*** |  |  |  |  |  |  |  |
| Aspirin | 1684/1684 | 452 (84.0%) | 684 (83.2%) | 263 (79.7%) | 0.188 | 280 (64.7%) |  |
| Clopidogrel | 1684/1684 | 354 (65.8%) | 519 (63.6%) | 203 (61.5%) | 0.430 | 190 (43.9%) |  |
| Tikagrelor | 1684/1684 | 72 (13.4%) | 119 (14.6%) | 46 (13.9%) | 0.822 | 56 (12.9%) |  |
| Prasugrel | 1684/1684 | 0 (0%) | 6 (0.7%) | 3 (0.9%) | 0.112 | 8 (1.9%) |  |
| ACE-inhibitors | 1684/1684 | 317 (58.9%) | 516 (63.2%) | 205 (62.1%) | 0.274 | 202 (46.7%) |  |
| A2R-blockers | 1684/1684 | 62 (11.5%) | 114 (14.0%) | 57 (17.3%) | 0.058 | 95 (21.9%) |  |
| Beta-receptor blockers | 1684/1684 | 447 (83.1%) | 687 (84.2%) | 269 (81.5%) | 0.538 | 277 (64.0%) |  |
| Statins | 1684/1684 | 448 (83.3%) | 680 (83.3%) | 269 (81.5%) | 0.739 | 271 (62.6%) |  |
| Dihydropyridines | 1684/1684 | 75 (13.9%) | 128 (15.7%) | 77 (23.3%) | 0.001 | 111 (25.6%) |  |
| Diuretics | 1684/1684 | 56 (10.4%) | 131 (16.1%) | 64 (19.4%) | 0.001 | 101 (23.3%) |  |
| Warfarin | 1684/1684 | 21 (3.9%) | 42 (5.2%) | 16 (4.9%) | 0.564 | 29 (6.7%) |  |
|  |  |  |  |  |  |  |  |

**Table S4:** Absolute numbers (%), event rates per 100 person-years and hazard ratios for all-cause mortality in 841 patients stratified by glucose perturbation group according to OGTT and HbA1c results (ADA criteria).

|  | **Total study population** | **Glycaemic status according to OGTT** | | | **Glycaemic status according to HbA1c** | | |  |
| --- | --- | --- | --- | --- | --- | --- | --- | --- |
|  |  | **Normal** | **Prediabetes** | **Diabetes** | **Normal** | **Prediabetes** | **Diabetes** |  |
|  | *(N=841)* | *(N=139)* | *(N=398)* | *(N=304)* | *(N=320)* | *(N=461)* | *(N=60)* |  |
| Number of events, N (%) | 108 (12.8) | 12 (8.6) | 46 (11.6) | 50 (16.4) | 35 (10.9) | 61 (13.2) | 12 (20.0) |  |
| Events/100 patient-years (95% CI) | 1.9 (1.5-2.4) | 1.4 (0.8-2.4) | 1.7 (1.3-2.2) | 2.3 (1.7-3.0) | 1.6 (1.2-2.3) | 1.9 (1.5-2.4) | 3.0 (1.7-5.3) |  |
|  |  |  |  |  |  |  |  |  |
| Unadjusted model |  | 1 (ref) | 1.14 (0.75-1.73) | 1.83 (0.95-3.53) | 1 (ref) | 1.14 (0.75-1.73) | 1.83 (0.94-3.56) |  |
| Age- and sex-adjusted model |  | 1 (ref) | 0.99 (0.53-1.87) | 1.27 (0.67-2.40) | 1 (ref) | 1.05 (0.69-1.59) | 1.66 (0.86-3.20) |  |
| CI = confidence interval, ref = reference category, N = number of patients | | | | |  |  |  |  |
|  |  |  |  |  |  |  |  |  |

**Figure S2**: Kaplan–Meier curve showing time to freedom from all-cause mortality for different categories of dysglycaemia according to A. Fasting and 2-hour post-load glucose results (OGTT) and B. HbA1c.

**Table S5:** Absolute numbers (%), event rates per 100 person-years and hazard ratios for the combined event (CE; first of myocardial infarction, hospitalisation for heart failure, ischaemic stroke or mortality) in 1684 patients stratified by glucose perturbation group according to OGTT (ADA criteria).

|  | **Total study population** | **Glycaemic status according to OGTT** | | |  |
| --- | --- | --- | --- | --- | --- |
|  |  | **Normal** | **Prediabetes** | **Diabetes** |  |
|  | *(N=1684)* | *(N=538)* | *(N=816)* | *(N=330)* |  |
| Number of events, N (%) | 739 (43.9) | 221 (41.1) | 364 (44.6) | 154 (46.7) |  |
| Events/100 patient-years (95% CI) | 8.5 (7.9-9.1) | 7.6 (6.6-8.6) | 8.9 (8.0-9.9) | 9.0 (7.7-10.6) |  |
|  |  |  |  |  |  |
| Unadjusted model |  | 1 (ref) | 1.14 (0.96-1.35) | 1.16 (0.94-1.42) |  |
| Age- and sex-adjusted model |  | 1 (ref) | 1.09 (0.92-1.28) | 1.09 (0.89-1.34) |  |
| CI = confidence interval, ref = reference category, N = number of patients | | | | |  |
|  |  |  |  |  |  |
